# Sexual Experience Promotes Adult Neurogenesis in the Hippocampus Despite an Initial Elevation in Stress Hormones

**DOI:** 10.1371/journal.pone.0011597

**Published:** 2010-07-14

**Authors:** Benedetta Leuner, Erica R. Glasper, Elizabeth Gould

**Affiliations:** Department of Psychology, Neuroscience Institute, Princeton University, Princeton, New Jersey, United States of America; Claremont Colleges, United States of America

## Abstract

Aversive stressful experiences are typically associated with increased anxiety and a predisposition to develop mood disorders. Negative stress also suppresses adult neurogenesis and restricts dendritic architecture in the hippocampus, a brain region associated with anxiety regulation. The effects of aversive stress on hippocampal structure and function have been linked to stress-induced elevations in glucocorticoids. Normalizing corticosterone levels prevents some of the deleterious consequences of stress, including increased anxiety and suppressed structural plasticity in the hippocampus. Here we examined whether a rewarding stressor, namely sexual experience, also adversely affects hippocampal structure and function in adult rats. Adult male rats were exposed to a sexually-receptive female once (acute) or once daily for 14 consecutive days (chronic) and levels of circulating glucocorticoids were measured. Separate cohorts of sexually experienced rats were injected with the thymidine analog bromodeoxyuridine in order to measure cell proliferation and neurogenesis in the hippocampus. In addition, brains were processed using Golgi impregnation to assess the effects of sexual experience on dendritic spines and dendritic complexity in the hippocampus. Finally, to evaluate whether sexual experience alters hippocampal function, rats were tested on two tests of anxiety-like behavior: novelty suppressed feeding and the elevated plus maze. We found that acute sexual experience increased circulating corticosterone levels and the number of new neurons in the hippocampus. Chronic sexual experience no longer produced an increase in corticosterone levels but continued to promote adult neurogenesis and stimulate the growth of dendritic spines and dendritic architecture. Chronic sexual experience also reduced anxiety-like behavior. These findings suggest that a rewarding experience not only buffers against the deleterious actions of early elevated glucocorticoids but actually promotes neuronal growth and reduces anxiety.

## Introduction

Negative or aversive stressful experiences are associated with deleterious consequences for the brain and behavior. Indeed, aversive stress and trauma are major predisposing factors for the development of psychopathology [1]. The hippocampus is particularly sensitive to aversive stress, responding with diminished adult neurogenesis [2], [3], [4], dendritic complexity [5], [6], [7] and synaptic plasticity [8]. Negative stress enhances anxiety [9], the regulation of which has been linked to the hippocampus [10]. Elevated glucocorticoid levels have been implicated in these effects – blocking stress-induced increases in corticosterone levels prevents detrimental effects on adult neurogenesis [4], dendritic complexity [6], and anxiety [9].

Despite evidence linking elevated stress hormones with impaired structural plasticity and hippocampal function, physical exercise increases glucocorticoid levels but is generally beneficial to health. Running increases glucocorticoid levels in rodents and humans [11], [12], while paradoxically enhancing structural plasticity, including adult neurogenesis [13], [14], dendritic spine density, and dendritic complexity [15], [16] in the hippocampus. Furthermore, exercise reduces anxiety [17] and improves learning and memory functions associated with the hippocampus [13]. Running also increases blood flow to the brain, improves cardiovascular fitness, and stimulates angiogenesis, all factors that could lead to improved neuronal growth and, ultimately, enhanced brain function [18]. However, a recent study showed that rewarding intracranial self-stimulation is sufficient to increase adult neurogenesis [19], suggesting that the hedonic aspect of physical exercise may be responsible for its beneficial effects on structural plasticity.

Exercise appears to be universally rewarding for rodents – rats and mice voluntarily run considerable distances if given access to a running wheel. Rats also develop place preferences for a running wheel [17], [20] and will bar press to gain access to running [21]. Collectively, these results suggest that the emotional valence or hedonic value of the stressor may play a role in determining whether an experience will produce negative or positive effects despite elevated glucocorticoid levels [22]. That is, positive or rewarding stress may buffer the brain from the potentially adverse consequences of physical exertion. To explore this possibility further, we examined whether sexual behavior, a natural rewarding experience, is associated with alterations in hippocampal structure, glucocorticoids, and anxiety.

## Materials and Methods

### Ethics Statement

Procedures were conducted in accordance with Princeton University AALAC (protocol # 1756, approved July 2009) and The National Institutes of Health Guide for the Care and Use of Laboratory Animals.

### Experimental Animals

Adult (>60 d of age) male and female Sprague-Dawley rats (Taconic, Germantown, NY) were provided unlimited access to food and water and maintained on a 12:12 light-dark cycle (lights on 1900 h). Males, housed 2-3/cage, were acclimated to the colony for ∼5 d. All rats within a cage were included in the same experimental group. Females were individually housed following bilateral ovariectomy under Nembutal anesthesia and allowed to recover for one week. Sexual receptivity was induced by subcutaneously injecting estrogen (200 ìg/0.2 ml sesame oil) and progesterone (500 ìg/0.2 ml sesame oil) 48 h and 4 h before the start of the experiment, respectively. Non-receptive females were ovariectomized but did not receive hormonal injections.

### Sexual experience

Male rats were placed in a novel cage with a sexually-receptive female, a non-receptive female, or were naive and remained undisturbed in their home cage. The same sexually-receptive females were used every 4^th^ day and as a result, the males on occasion were exposed to a female with which they had previously copulated. This did not change sexual behavior. Males were allowed to engage in sexual behavior for 30 min, starting from the first intromission. The duration of exposure to non-receptive females was matched to that of a sexually-experienced rat. Exposures were monitored and videotaped in the dark under red light illumination (1300–1600 h). For the long-term sexual experience studies, digital videos were analyzed for mounts, intromissions, and ejaculations [23].

### BrdU administration and perfusion

Male rats (n = 3–4/group) were exposed to a sexually-receptive female once (acute) and injected intraperitoneally with the DNA synthesis marker bromodeoxyuridine (BrdU) 30 min after the first intromission along with naive controls. The dose of BrdU was 200 mg/kg, which labels the maximal number of cells in the dentate gyrus [24]. All animals were perfused 2 h later. This post-BrdU survival time is sufficient to label cells in S-phase but not to allow the labeled cells to divide, thus providing a measure of cell proliferation. Additional cohorts of male rats were exposed to a sexually-receptive female once daily for 14 consecutive days (chronic). On the last day, rats were injected with BrdU 30 min following initiation of sexual behavior along with rats exposed daily to a non-receptive female. Also injected was an additional group of naive controls. All animals (n = 6–8/group) were perfused 2 h later. To determine whether chronic sexual experience affects adult neurogenesis, additional groups (n = 6–8/group) of sexually-experienced and naive rats were treated as described above but perfused 2 weeks later. The majority of new cells in the dentate gyrus express the mature neuronal marker NeuN 2 weeks post-BrdU labeling [24]. For perfusion, rats were anesthetized with Nembutal and transcardially perfused with 4% paraformaldehyde in 0.1 M phosphate buffer. Brains were post-fixed for at least 2 d.

### Corticosterone sampling and radioimmunoassay

The effect of acute and chronic mating experience on circulating corticosterone levels was measured in separate cohorts of male rats (n = 4–8/group) exposed to a sexually-receptive female once or once daily for 14 d. Rats were rapidly decapitated (between 1300–1600 hr) along with naive controls 30 min following initiation of sexual behavior. For chronic sexual experience, decapitations occurred on the last day of exposure to the sexually-receptive female. Trunk blood was collected, centrifuged (14,000 rpm), plasma obtained, and circulating levels of corticosterone measured by radioimmunoassay using a Coat-a-Count Rat Corticosterone kit (Siemens Healthcare Diagnostics, Plainfield, IN).

### Anxiety-like behavior

An additional cohort (n = 11/group) of naive and sexually-experienced rats were tested on the elevated plus maze and novelty suppressed feeding paradigms. For this experiment, daily exposure to sexual experience for 28 d was utilized in order to stimulate the production of a large pool of new neurons as well as to provide sufficient time for the new neurons to become incorporated into the existing circuitry. On the last day of sexual experience, rats were placed in the elevated plus maze that was located in a dimly lit room and consisted of a cross-shaped platform (height: 49.5 cm) with four arms (width: 10 cm; length: 110.5 cm), two of which were enclosed by walls 30.5 cm in height (San Diego Instruments, San Diego, CA). The rat was placed into the central area facing an open arm and allowed to explore for 10 min. The percentage of time spent on and number of entries into the open arms were used as measures of anxiety-like behavior. Locomotor activity was assessed using the number of closed arm entries. Afterwards, rats were returned to their home cage and food deprived. The following day, rats were again exposed to a sexually-receptive female and were tested on the novelty-suppressed feeding paradigm 24 h later. Rats were placed in one corner of a brightly lit open arena (58×42×35 cm), the floor of which was covered with ∼1.5 cm of clean bedding. The latency to chew a ∼2 g food pellet located in the center of the arena was recorded. If the rat did not begin feeding within 600 sec, the test was terminated and a latency of 600 sec was assigned. Immediately thereafter, rats were transferred to their home cage and the amount of food consumed by all rats within a cage in 300 sec was measured.

### Immunohistochemistry

Forty-µm thick coronal sections throughout the entire rostrocaudal extent of the dentate gyrus were cut with a Vibratome from half brains into a bath of 0.1 M phosphate buffered saline (PBS).

For BrdU and Ki67 peroxidase staining, a 1∶12 series of sections was mounted onto glass slides, dried, heated in 0.1 M citric acid, and rinsed in PBS. For BrdU, slides were then incubated in trypsin, rinsed, denatured in 2 M HCl:PBS, rinsed, and incubated in mouse monoclonal antibody against BrdU (1∶200; Vector Laboratories, Burlingame, CA) at 4°C. For Ki67, slides were incubated in 3% H_2_0_2_, rinsed, and incubated at room temperature in mouse monoclonal antibody against Ki67 (1∶100; Vector). The next day, slides were rinsed and reacted with a mouse ABC kit (Vector) followed by 0.01% diaminobenzidine with 0.003% H_2_O_2_ (Sigma-Aldrich, St. Louis, MO). Slides were counterstained with cresyl violet, dehydrated, cleared, and coverslipped under Permount (Fisher Scientific, Fair Lawn, NJ).

For assessment of cell phenotype, tissue from rats with a 2 week post-BrdU survival time was processed for double-labeling immunofluorescence for BrdU and the neuronal markers, TuJ1 or NeuN, or the astroglial marker, GFAP. Free-floating sections were denatured in 2 M HCl:TBS, rinsed in TBS, and incubated with rat anti-BrdU (1∶200; Accurate, Westbury, NY) plus mouse anti-TuJ1 (1∶500; Covance, Berkeley, CA), mouse anti-NeuN (1∶500; Chemicon, Temecula, CA), or guinea pig anti-GFAP (1∶1000; Advanced Immunochemical, Long Beach, CA) for 2 d at 4°C. Sections were then rinsed, incubated with biotinylated anti-rat (Chemicon), rinsed, and incubated with streptavidin-conjugated Alexa 568 (1∶1000; Molecular Probes, Eugene, OR) for BrdU, and with goat anti-mouse or goat anti-guinea-pig Alexa 488 (1∶500; Molecular Probes) for TuJ1, NeuN, or GFAP. After rinsing, sections were mounted onto slides and coverslipped using glycerol:TBS (3:1).

### Golgi impregnation

The other brain half from naive rats and those with 14 d of sexual experience perfused 2 h after BrdU injection were processed using a modified Golgi-Kopsch protocol. Small tissue blocks containing the hippocampus were incubated in 4% potassium dichromate for 3 d in the dark. The solution was changed daily. Next, the blocks were rinsed in graded concentrations of silver nitrate (0.25. 0.50, 0.75, 1%; 5 min each), then shaken in 1% silver nitrate for 7 d in the dark. Unilateral coronal sections were cut with a Vibratome (125 ìm thick) in distilled water, rinsed in graded concentrations of ethanol, cleared, mounted on slides, and coverslipped under Permount.

### Microscopic data analysis

Slides were coded prior to data collection. The code was broken after analyses were complete. Labeled cells in the granule cell layer, subgranular zone, and hilus were counted at 100× on an Olympus BX-50 light microscope. Counts were multiplied by 24 to obtain estimates of BrdU- or Ki67-labeled cells in the dentate gyrus per brain.

For BrdU immunofluorescence, the percentage of BrdU-labeled cells in the granule cell layer and subgranular zone expressing NeuN, TuJ1, or GFAP was determined using a Zeiss Axiovert confocal laser scanning microscope (510 LSM; lasers, Argon 458/488 and HeNe 543; Zeiss, Oberkochen, Germany). For each brain and each marker, 25 randomly selected BrdU-labeled cells were analyzed. Optical stacks of 1-ìm thick sections were obtained through putatively double-labeled cells. To verify double labeling throughout their extent, cells were examined in orthogonal planes.

For each brain, dendritic spine density was analyzed on 5 randomly selected dendritic segments (10–20 ìm long) on 5 dentate gyrus granule neurons and 5 CA1 pyramidal neurons at 100× using an Olympus BX-60 microscope equipped with a motorized stage and camera linked to a computer with Neurolucida software (Microbrightfield, Williston, VT). Golgi-impregnated neurons were selected for analysis if they were thoroughly impregnated and sufficiently isolated from surrounding cells. For every cell, dendritic segments were on secondary or tertiary dendrites, located at least 20 ìm from the soma, and mostly in one focal plane. Only spines extending away from the shaft were counted.

For dendritic length and branching analyses of dentate gyrus granule cells, 5 cells/animal were selected from the set of neurons satisfying the criteria described above. These cells were traced using the 40× objective providing an average dendritic tree length. Branch points were counted when a dendrite exhibited a distinct bifurcation.

### Statistics

Data were analyzed using unpaired Student's t-tests or ANOVA followed by Newman-Keuls post-hoc analysis, where appropriate. Welch's correction for unequal variance was applied when necessary.

## **Results**

### Sexual behavior

Male rats readily engaged in sexual behavior when exposed to a sexually-receptive female. For the acute sexual experience study, all rats copulated during their single exposure to the receptive female. For the chronic sexual experience experiments, all rats copulated on numerous days but some did not do so every day despite exposure to a sexually-receptive female. All rats were included in the analysis and the mean percent copulation for a two week exposure was over 75% (Table 1).

**Table 1:** Sexual behavior on day 1, 7, and 14 of male rats (n = 6) exposed to a sexually-receptive female daily for 14 consecutive days.

	Day of sexual experience		
	Day 1	Day 7	Day 14
**Average number of mounts**	33.33±10.35	38.33±17.99	26.83±6.80
**Average number of intromissions**	28.83±7.56	31.17±11.59	63.33±13.95
**Average number of ejaculations**	1.00± 0.37	1.00± 0.45	1.67±0.21

There was no difference across days in the average number of mounts, intromissions, or ejaculations displayed during a 30 min exposure to a hormonally-primed sexually receptive female. Numbers represent mean ± SEM. *P*>0.05 for all comparisons, repeated measures ANOVA.

### Effects of acute sexual experience on corticosterone levels and cell proliferation

**Figure 1:**
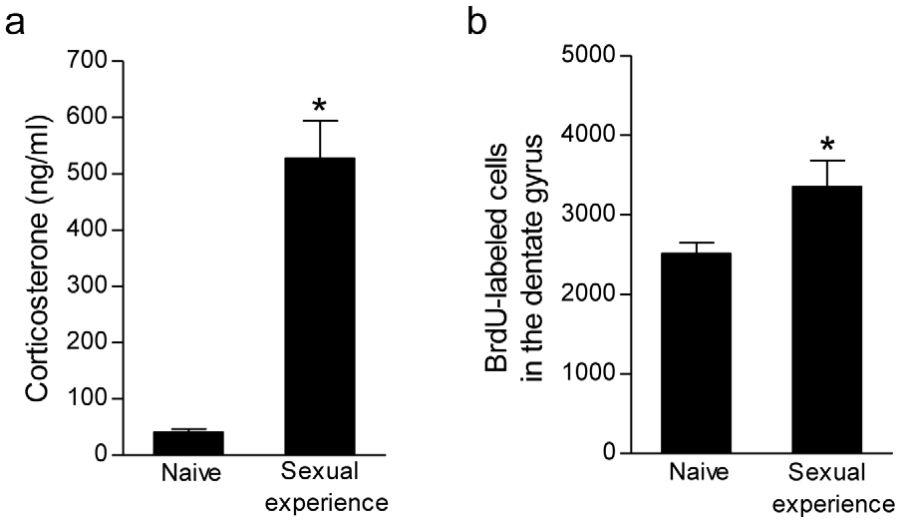
Acute sexual experience enhances cell proliferation in the dentate gyrus despite elevated glucocorticoid levels. Compared to naive controls, a single sexual experience elevated (a) corticosterone levels and (b) the number of proliferating cells (labeled with BrdU and examined after a 2 h survival). Bars represent mean ± SEM, ^*^ *P*<0.05.

Circulating corticosterone levels were elevated in male rats after sexual experience compared to naive controls (t_3_ = 7.30, *P*<0.005; Fig.1a). Despite increased corticosterone levels, a single bout of sexual experience was sufficient to increase cell proliferation in the dentate gyrus. Males with 30 min of sexual experience, injected with BrdU, and perfused 2 h later exhibited a significant increase in BrdU-labeled cells compared to naive controls (t_5_ = 2.64, *P*<0.05; Fig.1b).

### Effects of chronic sexual experience on corticosterone levels, cell proliferation, and neurogenesis

**Figure 2:**
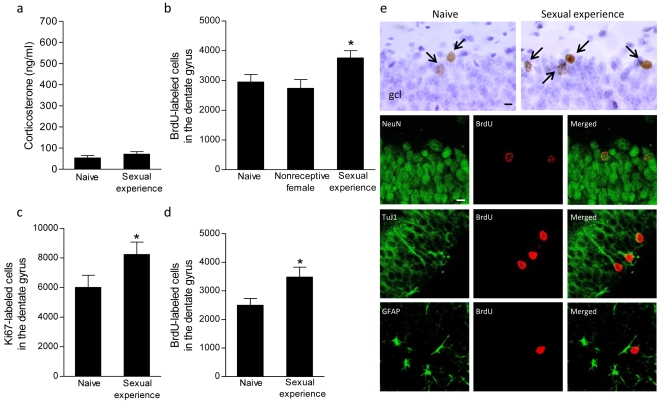
Chronic sexual experience enhances cell proliferation and adult neurogenesis without altering glucocorticoid levels. (a) 14 days of sexual experience did not increase glucocorticoid levels compared to naive controls. (b) The number of proliferating cells (those labeled with BrdU and examined after a 2 h survival) was increased in rats with chronic sexual experience compared to naive controls or those exposed to a non-receptive female. (c) The increased number of proliferating cells in sexually-experienced rats was confirmed using the endogenous marker of cell proliferation, Ki67. (d, e) Sexual experience produced more new neurons (those labeled with BrdU and examined after a 2 week survival) ― the majority of BrdU-labeled cells (arrows) expressed TuJ1 and NeuN, but not GFAP. Scale bars, 10 µm. gcl, granule cell layer. Bars represent mean ± SEM, ^*^ *P*<0.05.

Circulating corticosterone levels were no longer elevated in male rats after 14 d of sexual experience (t_14_ = 1.08, *P* = 0.30; Fig.2a). However, chronic sexual experience continued to enhance cell proliferation in the dentate gyrus. Male rats exposed to a sexually-receptive female daily for 14 d had more proliferating cells (BrdU-labeled cells after a 2 h post-BrdU survival time) than rats exposed to a non-receptive female and naive controls (F_2,19_ = 3.60, *P*<0.05; Fig.2b). Since no differences existed between naive rats and those exposed to a non-receptive female (*P*>0.05), the latter group was excluded from remaining comparisons. Analysis of Ki67, an endogenous marker of cell proliferation, revealed a similar change – 14 d of sexual experience increased the number of Ki67-labeled cells compared to controls (t_19_ = 1.89, *P*<0.05; Fig.2c).

Chronic sexual experience also enhanced adult neurogenesis in the dentate gyrus. Males with 14 d of mating experience, BrdU injections on the last day, and perfusion 2 weeks later had more BrdU-labeled cells compared to naive controls (t_12_ = 2.18, *P*<0.05; Fig.2d,e). Most (∼75%) BrdU-labeled cells expressed the neuronal markers, NeuN and TuJ1. A small percentage (∼5%) expressed the astroglial marker, GFAP. There was no difference in the proportion of BrdU-labeled cells expressing these phenotypic markers across groups (*P* values >0.05) suggesting that the mating-induced increase in BrdU-labeled cells represents an increase in neurogenesis.

### Effects of sexual experience on dendritic architecture in the hippocampus

**Figure 3:**
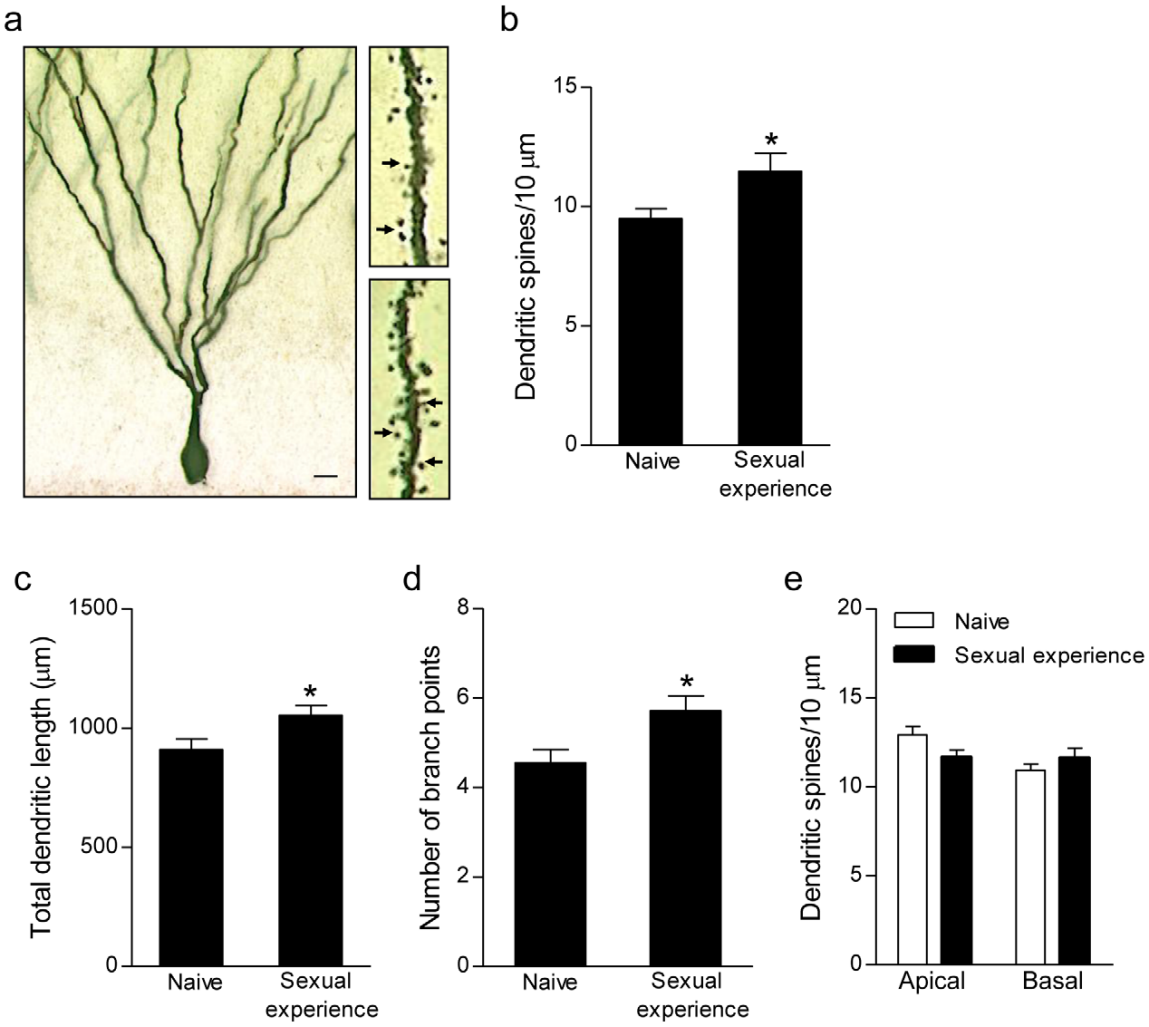
Sexual experience alters dendritic morphology. (a) Golgi-impregnated dentate gyrus granule neuron with high magnification images of dendritic segments from a naive (top) and a sexually-experienced (bottom) rat. Arrows indicate dendritic spines. Scale bar, 10 µm (cell), 1 µm (dendrite). Sexual experience increased (b) dendritic spine density, (c) dendritic length and, (d) dendritic branching of granule neurons. (e) Dendritic spine density on apical and basal dendrites of CA1 pyramidal neurons was unaffected. Bars represent mean ± SEM, ^*^ *P*<0.05.

Fourteen days of sexual experience enhanced the number of dendritic spines on dentate gyrus granule neurons (t_12_ = 2.44, *P*<0.05; Fig.3a,b). In addition, mating increased total dendritic length (t_14_ = 2.34, *P*<0.05; Fig.3c) and the number of branch points (t_14_ = 2.59, *P*<0.05; Fig.3d) of granule neurons. The increase in dendritic spine density concomitant with an overall increase in dendritic length and branching suggests that the change in spine density reflects an overall increase in spine number, rather than an increase in spine density due to dendrite shrinkage. By contrast, CA1 pyramidal neurons were unaffected (Fig.3e).

### Effects of sexual experience on anxiety-like behavior

**Figure 4:**
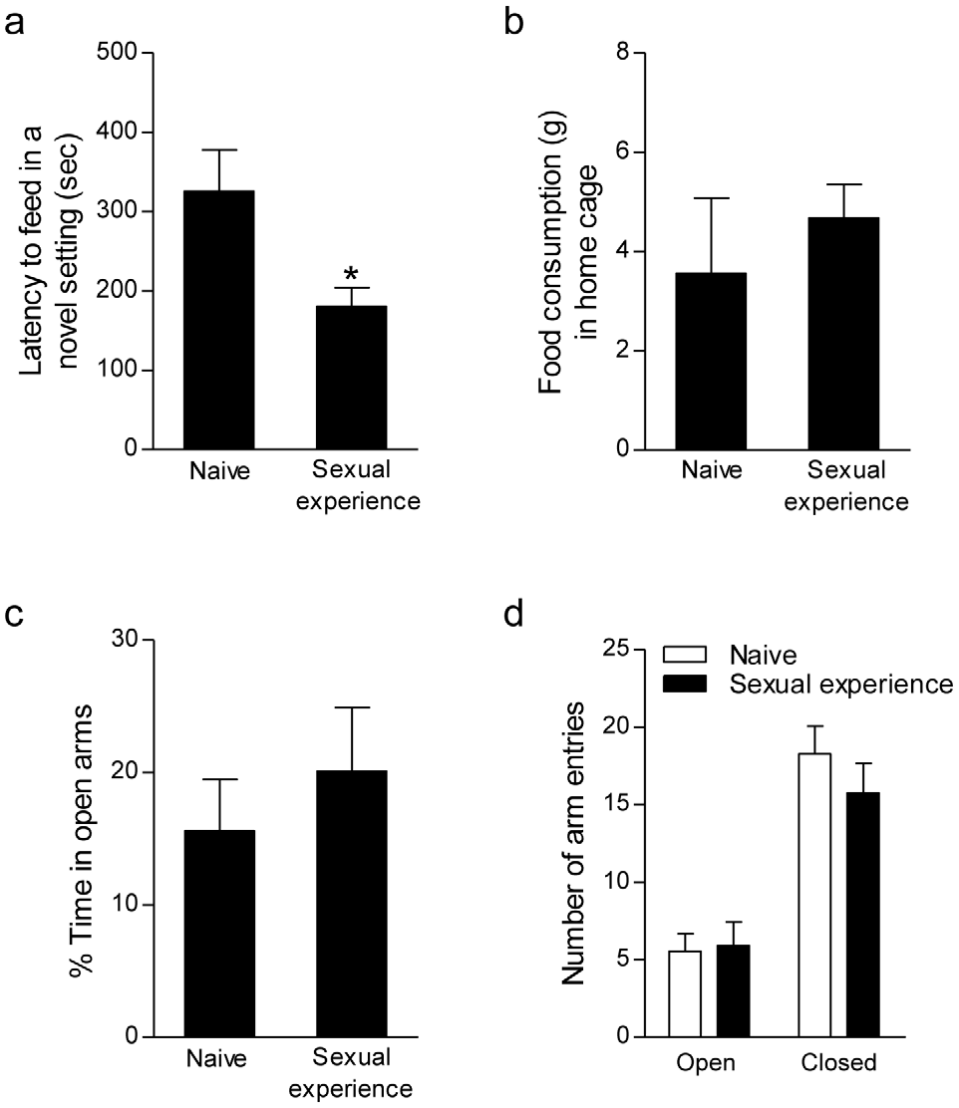
Sexual experience reduces some measures of anxiety-like behavior. (a) Compared to naive controls, sexual experience reduced anxiety-like behavior on the novelty suppressed feeding paradigm. (b) Home cage food consumption was unaltered. In contrast, anxiety-like behavior in the elevated plus maze was unaffected by sexual experience. (c) Percent time in the opens arms as well as (d) open and closed arm entries were similar in naive and sexually-experienced rats. Bars represent mean ± SEM, ^*^ *P*<0.05.

Sexually-experienced rats were less anxious than naive controls when tested on the novelty suppressed feeding paradigm, as indicated by a shorter latency to consume a familiar food in a novel environment (t_13_ = 2.52, *P*<0.05; Fig.4a). There was no difference in home cage food consumption between groups (*P*>0.05; Fig.4b) suggesting that the effect of sexual experience was not due to group alterations in hunger or motivation. In the elevated plus maze, no changes were observed between naive and sexually-experienced rats in the percent time spent in the open arms, open arm entries, or closed arm entries (Figure 4c,d).

## Discussion

Our results indicate that acute sexual experience enhances cell proliferation in the dentate gyrus of adult male rats despite substantial elevations in glucocorticoid levels. Furthermore, chronic sexual experience enhances cell proliferation, adult neurogenesis, and the number of dendritic spines in the dentate gyrus of adult male rats. Anxiety-like behavior, as assessed by novelty suppressed feeding, a paradigm that has been linked to neuronal growth in the hippocampus [25], was also reduced by chronic sexual experience.

Elevated glucocorticoid levels have long been associated with detrimental effects on hippocampal structure and function. Experimental increases in corticosterone levels result in dendritic atrophy, suppressed cell proliferation, and reduced adult neurogenesis [26], [27]. Moreover, exposure to aversive stressors, like predator odor, cold water, and restraint, have effects on hippocampal structure that are comparable to those of raising glucocorticoids pharmacologically [2], [3], [4], [5], [14]. Similarly, aversive stress or pharmacological elevation in glucocorticoid levels enhances anxiety and impairs some hippocampal learning and memory functions [8], [9], [28]. The link between elevated glucocorticoid levels and decrements in hippocampal structure and function has been established in multiple studies – preventing stress-induced increases in adrenal steroids inhibits the negative actions of stress on neurogenesis [4], dendritic atrophy [6], anxiety [9], and learning [29]. Consideration of this large body of literature in the context of the present results raises an obvious contradiction – despite elevated glucocorticoid levels [30], [31], the hippocampus responds positively to acute sexual experience with enhanced cell proliferation. The specific characteristics of stressful experiences that determine the structural and physiological outcome for the hippocampus remain an open question. One possibility is that the emotional valence of the stressor plays an important role.

Sexual experience is a strong motivator for rodents [32]. Male rats learn place preferences to mating [33] and will bar press to gain access to a sexually-receptive female [34]. The rewarding aspect of mating shares features in common with running, an experience that also raises glucocorticoid levels [11], [12] and promotes neuronal growth [13], [14], [15], [16]. Together with recent work demonstrating that rewarding brain stimulation enhances adult neurogenesis [19], these findings suggest that the typically deleterious actions of elevated glucocorticoid levels can be overridden if the stressful experience has hedonic value. This possibility raises questions about what factors associated with reward might participate in protecting the brain from the negative influence of stress hormones and further, whether similar or other factors are responsible for the beneficial effects of sexual experience. In this regard, a role for neuromodulators altered with sexual experience and known to influence adult neurogenesis, like opiates [35] or dopamine [36], seem plausible, as does oxytocin, a neuropeptide that buffers the brain and body against some of the adverse consequences of stress hormones [37].

Unlike acute sexual experience, chronic exposure to a sexually-receptive female for 14 d did not increase corticosterone levels. Elevations in corticosterone levels have been reported in male rats up to 3 d following sexual behavior [30], [38] indicating that corticosterone responses become habituated sometime between 3 d and 14 d of sexual experience. Despite differing hormonal responses to sexual experience, both acute and chronic sexual experience was associated with an enhancement in cell proliferation. Other work has shown that 5 d of sexual experience can stimulate cell proliferation but only in rats exposed to aversive stress [39].

Chronic sexual experience also enhanced adult neurogenesis in the dentate gyrus. Mating induced-increases in neurogenesis have been reported in the olfactory bulb of female mice, but not in the dentate gyrus [40]. However, exposing female mice to the pheromones of dominant males is sufficient to enhance neurogenesis in both the olfactory bulb and hippocampus [41]. The specific aspects of the mating encounter that drive changes in hippocampal neurogenesis in male rats have yet to be determined. It is possible that full copulation is necessary or that odor and other sensory cues arising from sexually-receptive females are involved. Each of these is known to be rewarding for male rodents [32], [33], [34] raising the possibility that the reward value inherent to sexual behavior is what ultimately mediates the beneficial effects of sexual experience on hippocampal neurogenesis.

In contrast to the hippocampus and olfactory bulb, reproductive activity does not alter the proliferation or survival of new cells in the medial nucleus of the amygdala or medial preoptic area of male hamsters, brain regions that are part of the mating circuitry [42]. However, since these are areas where the rate of neurogenesis is low [43], it may be difficult to detect changes. Nonetheless, brain regions that mediate sexual behavior do undergo morphological alterations in the form of dendritic and synaptic remodeling [44], [45]. Similarly, we found that chronic sexual experience enhanced dendritic spine density and dendritic complexity of dentate gyrus granule neurons.

Numerous studies have shown that sexual experience exerts anxiolytic effects in a variety of paradigms including the elevated plus maze, open field, elevated zero maze, light-dark box, and conditioned defensive burying [46], [47], [48]. Here, we extend these findings to include a reduction in anxiety on the novelty suppressed feeding paradigm following sexual experience. However, we did not detect alterations in anxiety following sexual experience on the elevated plus maze. These discrepancies may be related to differences in the duration of sexual experience across studies or possible variations in the testing conditions that are known to influence behavior in the elevated plus maze [49]. Nonetheless, a positive relationship between the number of BrdU-labeled cells and a reduction in anxiety on the novelty suppressed feeding paradigm, a behavior that has been linked to this brain region [10], raises the possibility that the two changes are linked. Some evidence suggests that changes in the number of new neurons in the dentate gyrus result in altered anxiety. For example, depletion of new neurons enhances anxiety-like behavior [50] and prevents some anxiolytic actions of antidepressants [25]. Our results suggest that the sexual experience-induced increase in adult neurogenesis contributes to the reduction in anxiety, but additional interpretations must be considered. Other structural changes, such as alterations in dendritic spine density or complexity might be responsible for such functional effects, either solely or together with changes in new neurons. It's also possible that biochemical or physiological changes that occur following acute sexual experience that are completely independent of structural change may be responsible for the effects on anxiety. Additional time course studies, such as those that provide chronic sexual experience followed by a period of no sexual experience, will be needed to rule out this possibility.

Numerous studies have linked experience to changes in adult neurogenesis, dendritic spines, and hippocampal function. In general, stress effects have been associated with negative outcomes in terms of brain structure and function, and elevated glucocorticoid levels seem to be mechanistically involved. The results of the present study, combined with the existing literature, suggest that naturally rewarding experiences are not only beneficial for the brain but also suggest that the hedonic value of an experience plays a more important role than glucocorticoid levels in determining whether the outcome will be beneficial or detrimental. The extent to which fundamentally aversive experiences can be recast as positive through learning, thus minimizing their damaging influence, remains to be determined.
